# Human SR-BII mediates SAA uptake and contributes to SAA pro-inflammatory signaling *in vitro* and *in vivo*

**DOI:** 10.1371/journal.pone.0175824

**Published:** 2017-04-19

**Authors:** Irina N. Baranova, Ana C. P. Souza, Alexander V. Bocharov, Tatyana G. Vishnyakova, Xuzhen Hu, Boris L. Vaisman, Marcelo J. Amar, Zhigang Chen, Alan T. Remaley, Amy P. Patterson, Peter S. T. Yuen, Robert A. Star, Thomas L. Eggerman

**Affiliations:** 1 Department of Laboratory Medicine, Clinical Center, National Institutes of Health, Bethesda, Maryland, United States of America; 2 Renal Diagnostics and Therapeutics Unit, National Institute of Diabetes and Digestive and Kidney Diseases, National Institutes of Health, Bethesda, Maryland, United States of America; 3 National Heart, Lung and Blood Institute, National Institutes of Health, Bethesda, Maryland, United States of America; 4 Division of Diabetes, Endocrinology and Metabolic Diseases, National Institute of Diabetes and Digestive and Kidney Diseases, National Institutes of Health, Bethesda, Maryland, United States of America; University of Navarra School of Medicine and Center for Applied Medical Research (CIMA), SPAIN

## Abstract

Serum amyloid A (SAA) is an acute phase protein with cytokine-like and chemotactic properties, that is markedly up-regulated during various inflammatory conditions. Several receptors, including FPRL-1, TLR2, TLR4, RAGE, class B scavenger receptors, SR-BI and CD36, have been identified as SAA receptors. This study provides new evidence that SR-BII, splice variant of SR-BI, could function as an SAA receptor mediating its uptake and pro-inflammatory signaling. The uptake of Alexa Fluor488 SAA was markedly (~3 fold) increased in hSR-BII-expressing HeLa cells when compared with mock-transfected cells. The levels of SAA-induced interleukin-8 secretion by hSR-BII-expressing HEK293 cells were also significantly (~3–3.5 fold) higher than those detected in control cells. Moderately enhanced levels of phosphorylation of all three mitogen-activated protein kinases, ERK1/2, and p38 and JNK, were observed in hSR-BII-expressing cells following SAA stimulation when compared with control wild type cells. Transgenic mice with pLiv-11-directed liver/kidney overexpression of hSR-BI or hSR-BII were used to assess the *in vivo* role of each receptor in SAA-induced pro-inflammatory response in these organs. Six hours after intraperitoneal SAA injection both groups of transgenic mice demonstrated markedly higher (~2-5-fold) expression levels of inflammatory mediators in the liver and kidney compared to wild type mice. Histological examinations of hepatic and renal tissue from SAA-treated mice revealed moderate level of damage in the liver of both transgenic but not in the wild type mice. Activities of plasma transaminases, biomarkers of liver injury, were also moderately higher in hSR-B transgenic mice when compared to wild type mice. Our findings identify hSR-BII as a functional SAA receptor that mediates SAA uptake and contributes to its pro-inflammatory signaling via the MAPKs-mediated signaling pathways.

## Introduction

Serum amyloid A (SAA) is a 12-14-kDa highly conserved acute phase apolipoprotein that is predominantly secreted by hepatocytes. Normally present in plasma in only trace amounts, SAA is a major acute phase reactant, whose plasma levels may increase up to 1000-fold [1,2] reaching serum concentrations of up to 80 μM in response to various insults, including trauma, infection, inflammation, and neoplasia, indicating its critical role in host defense mechanisms [3]. While the majority of SAA is found in association with high-density lipoproteins, up to 15% of SAA exists in a lipid-free or lipid-poor form [2]. Unlike other acute phase proteins, which are synthesized primarily in the liver, acute phase SAA (A-SAA) is also markedly expressed at local sites of tissue inflammation. In humans, the expression and production of A-SAA have been found in several cell types within atherosclerotic lesions, including endothelial cells, macrophages, adipocytes, and smooth muscle cells [4] as well as in the epithelial cells of several normal tissues [5].

In addition to its well-established acute response to inflammatory stimuli, SAA elevation can also be observed in multiple chronic inflammatory conditions, such as secondary amyloidosis [6], atherosclerosis [2,7], inflammatory bowel disease [8], rheumatoid arthritis [9,10] and chronic kidney disease [11]. Increased SAA plasma levels were also found in patients with obesity [12,13], insulin resistance [14], metabolic syndrome [15], and diabetes type 2 [12,16].

Multiple studies suggest that SAA may have profound effects on innate immunity as a result of its chemotactic and cytokine-inducing activities. A-SAA induces the secretion of pro-inflammatory cytokines tumor necrosis factor-α (TNF-α), interleukin-1β (IL-1β), and interleukin-8 (IL-8) [17], and acts as a chemoattractant for human monocytes, neutrophils and T cells [18,19]. Another recent study provided evidence for SAA as a potent activator of the NLRP3 inflammasome, demonstrating SAA as a mediator, providing signals needed for expression of pro–IL-1β and activation of the inflammasome cascade, resulting in activation of caspase-1 and secretion of mature IL-1β [20].

The diverse effects suggest that SAA may interact with more than one receptor and activate multiple signaling pathways. Earlier studies revealed several proteins that are capable of binding and/or mediating various SAA functions. FPRL1 (formyl peptide receptor like-1) protein was shown to mediate SAA–induced chemotactic migration of leukocytes [21] as well SAA cytokine–inducing activity in various phagocytic cells, including human neutrophils [22] and monocytes [23]. The scavenger receptor SR-BI has been demonstrated to mediate the cholesterol transport of HDL-associated SAA [24], whereas its human orthologue CLA-1 has been shown to internalize and mediate the pro-inflammatory activity of lipid-poor SAA via MAPK signaling pathways [25]. More recent experimental evidence suggests that toll-like receptors (TLRs) could also function as SAA receptors, mediating its signaling in macrophages. TLR2 has been demonstrated to bind SAA and mediate SAA-induced pro-inflammatory cytokine expression in bone marrow-derived macrophages [26] and activation of NLRP3 inflammasome in dendritic cells [27], while TLR4 was shown to be required for SAA-induced NO production through the activation of ERK1/2 and p38 MAPKs in peritoneal macrophages [28].

SR-BI, its splice variant SR-BII, and CD36 are members of the scavenger receptor family class B, that have high structural homology and all localize in plasma membrane caveolae-like domains which facilitate lipid exchange and cell signaling [29]. These receptors also share ligands, including native and modified lipoproteins [30,31] anionic phospholipids [32], amphipathic α-helical peptides [33–35], various bacteria [35–40] and bacterial products, such as LPS and cpn60 [35,41].

Our previous studies demonstrated SAA binding to and signaling through the CLA-1 (CD36 and LIMPII analogous-1), human orthologue of rodent SR-BI [25], and CD36 [42] via the MAPK kinase signaling pathways in epithelial cell lines overexpressing these receptors. In addition, more recent studies provided further evidence of a pathophysiological role of A-SAA in promoting the pro-inflammatory response in rheumatoid arthritis (RA) through SR-BI [43].

Despite serum amyloid A proteins being well-recognized markers of sepsis, and multiple reports demonstrated SAA presence at various inflammation sites [6–11], its own pathogenic role in acute inflammation and tissue injury during endotoxemia/sepsis remains poorly investigated. It was previously reported that in animal models of lethal endotoxemia recombinant SAA exacerbated endotoxemic lethality, significantly reducing survival rates. At the same time in a clinically relevant animal model of CLP-induced sepsis, repetitive administration of SAA-neutralizing immunoglobulins resulted in significant improvement of animal survival rates [44].

Our most recent studies that used transgenic mice overexpressing human SR-BI and SR-BII revealed that hSR-BII, and to a lesser extent hSR-BI, have a major contribution to the LPS-induced pro-inflammatory response and organ injury in a model of non-lethal endotoxemia [45]. Considering that all three SR-BI, SR-BII and CD36 scavenger receptors share a wide set of ligands including those with amphipathic α-helical domains [33,35] we suggested that SR-BII, like two other SR-B family members, could be a potential receptor involved with binding and pro-inflammatory signaling of SAA, an amphipathic protein with two amphipathic α-helices in its molecule. In this study we used *in vitro* and *in vivo* gain-of-function models—human cell lines overexpressing human SR-BI and SR-BII and transgenic mice with pLiv-11-directed liver/kidney overexpression of these two receptors [45]. This approach allowed us to investigate the individual role of each receptor in SAA-induced uptake and pro-inflammatory signaling *in vitro*, as well as in inflammation and tissue damage *in vivo*.

Findings of this study demonstrate that hSR-BII is a functional SAA receptor that mediates its uptake and contributes to SAA-induced pro-inflammatory signaling. Our data suggests that similar to previously reported hSR-BI- and CD36-dependent signaling of SAA, its signaling *via* hSR-BII might also involve MAPKs-mediated pathways. Additionally, the results of our *in vivo* experiments indicate that both hSR-BI and hSR-BII contribute to SAA-mediated organ injury and local tissue inflammation.

## Materials and methods

### Reagents

Recombinant synthetic human apo-SAA was purchased from PeproTech. The lipid content of the recombinant apo-SAA was analyzed by the phospholipid B enzymatic method (Wako, Richmond, VA), and the cholesterol content was determined by an enzymatic cholesterol method on a Cobas Fara II analyzer (Roche Applied Science). These assays indicated that the SAA preparation contained only small amounts of phospholipids (<5 ng/μg) and cholesterol (<2 ng/μg) and hence was considered a lipid-poor form of SAA throughout this study. The synthetic amphipathic peptides were synthesized by a solid-phase procedure as previously reported [34]. All reagents used for RNA isolation, reverse transcription and real-time PCR were from Life Technologies. Enzyme-linked immunosorbent assay (ELISA) kits for quantifying mouse IL-6, IL-1β and CXCL1 and human IL-8 were from Life Technologies and for mouse MIP-2 from R&D Systems. A competitive ELISA kit for quantifying corticosterone was from Enzo Life Sciences and a kit for colorimetric assay of nitrate (NOx) was purchased from Cayman Chemical. Anti-human SR-BI/BII antibody was from BD Biosciences (cat. # 610883), rabbit anti-human SR-BI and anti-human SR-BII antibody were custom produced against C-terminal domain specific peptides of hSR-BI (CTSAPKGSVLQEAKL, Anaspec, San Jose, CA), or hSR-BII (CLPDSPSRQPPSPTA, Sigma, St. Louis, MO). Custom antibodies were validated in Western blotting assay using cell lysates from HeLa cell line stably transfected with hSR-BI and hSR-BII [45]. Anti-mouse β-actin antibody, alkaline phosphatase secondary antibody and cholesterol quantitation kit were from Sigma Aldrich. Antibodies against phosphorylated and non-phosphorylated forms of MAP kinases (ERK1/2, SAPK/JNK and p38) were purchased from Cell Signaling Technology, Inc. MAPK inhibitors—UO126 (selective MAP2K inhibitor) and PD90859 (MAP2K/MEK inhibitor), SB202190 (highly selective p38 inhibitor) and SP600125 (selective JNK inhibitor) were purchased from Tocris Bioscience.

### Mice and cell culture

The liver-specific expression vector pLiv-11, which contains the human apoE promoter [46] was used to express SR-BI in the liver. Full-length (1.7-kb) human SR-BI (hSR-BI) cDNA (GenBank: BC112037.1) was flanked by Not I linkers and inserted into a unique Not I site of modified pLIV.11. Clones with the correct orientation of the transgene were selected after digestion of the plasmid DNA by Sph I and Aat II. The resulting pLiv-11- hSR-BI plasmid was digested with Sal I and Spe I, and an 11.6-kb DNA fragment LIV-hSR-BI, containing the complete expression cassette was isolated, purified and used for generating the transgenic mice (C57BL/6J). The LIV-hSR-BII construct was created the same way, by using the human SR-BII gene [47].

Mice were kept at the NIH animal facility under specific pathogen free conditions. All animal studies were approved by the Animal Care and Use Committee (ACUC) of the NHLBI under protocols H-0050R2 and H-0100R2 or NIDDK ACUC under protocol K100-KDB-15. The mice were monitored immediately after intervention, then after one and three hours to ensure that mice were not ill. Criteria for premature euthanasia were based on a points system of clinical scoring, where animals with a score exceeding 5 would be euthanized immediately. Points were scored as follows: depressed respiratory rate (2), apneustic respiration (5), spontaneous activity without stimulus (0), activity in response to tactile stimuli (1) delayed activity in response to tactile stimuli (2) unresponsive to tactile stimuli (5) piloerection (1) and lack of eye grooming (1). All mice had a score of 0 throughout the experiment.

*In vivo* studies were performed as follows: 11–12 week old male wild-type (WT), hSR-BI tgn or hSR-BII tgn mice were injected intraperitoneally (IP) with SAA (2 mg/kg) or PBS (using same volume, approximately 150 μl per mouse; n = 3 for PBS-treated and n = 3–5 for SAA-treated groups). None of the mice had a score above zero. Six hours after SAA/PBS injection, mice were anesthetized by ketamine/xylazine/acepromazine (80/10/0.02 mg/kg, IM), then blood and organs were collected, and mice were euthanized by exsanguination.

Wild-type HeLa cells were transfected with human SR-BI and SR-BII expressing pcDNA 3.1 plasmids by using the lipofectamine reagent and further selected in the presence of 800 μg/ml G418. Human embryonic (epithelial) kidney cells (HEK293, ATCC) were also stably transfected to express hSR-BI and hSR-BII, respectively) as described previously [38,41].

### Alexa 488—Labelled ligands uptake and competition experiments

Human apolipoprotein E-free high density lipoproteins (HDL) were isolated from the plasma of healthy donors as previously reported [38]. HDL, L37pA and L3D-37pA peptides and SAA were conjugated with Alexa Fluor 488, using a protein labeling kit (Invitrogen) following the vendor’s instructions.

All incubations were performed in Dulbecco's modified Eagle's medium containing 0.1% bovine serum albumin at 37°C. Uptake experiments with HeLa cells were performed using Alexa 488–labeled ligands at concentrations between 2.5 and 40 μg/ml, in triplicate, in a 96-well plate. After 2 hours of incubation the cells were rinsed 3 times with ice-cold PBS and read in a fluorescence plate reader (Wallac Victor 1420 Multilabel Counter, PerkinElmer Life Sciences). Competition experiments were performed using 5 μg/ml of Alexa 488-SAA and unlabeled ligands ranging in concentration from 0 to 125 μg/ml. Following 2-hour incubation and washing with ice-cold PBS, cell-associated fluorescence was analyzed by a fluorescence plate reader.

### Total RNA isolation and quantitative real-time PCR analysis

For RNA isolation, tissue samples preserved in RNAlater stabilization solution, were homogenized in TRIzol Reagent (Precellys 24, Bertin Technologies). RNA was isolated with the PureLink RNA

Mini Kit after DNase treatment. RNA (2 μg) was reverse-transcribed using a TaqMan Reverse Transcriptase Reagent Kit. Real-time qPCR assays were performed with a StepOne Real-Time

PCR System (Applied Biosystems), using 40 ng of cDNA per reaction. A list of TaqMan Gene Expression assays used in the study is shown in Table 1.

**Table 1:** TaqMan Real-Time PCR assays used in the study.

Species	Gene Name	Gene Symbol	Life Technologies ID number
Mouse	Interleukin 1 beta	Il1b	Mm00434228_m1
Mouse	Interleukin 6	Il6	Mm00446190_m1
Mouse	Chemokine (C-X-F motif) ligand 1	Cxcl1	Mm04207460_m1
Mouse	Chemokine (C-C motif) ligand 2	Ccl2	Mm00441242_m1
Mouse	Tumor necrosis factor	Tnfa	Mm00443258_m1
Mouse	NLR family, pyrin domain containing 3	Nlrp3	Mm00840904_m1
Mouse	CD68 antigen	Cd68	Mm03047343_m1
Mouse	Glyceraldehyde-3-phosphate dehydrogenase	Gapdh	Mm03302249_g1

Relative levels of gene expression were measured by the comparative CT (ΔΔCT) method with mouse β-actin or GAPDH genes as reference genes. All gene expression results were analyzed using the 2-∆∆CT formula and presented as normalized fold changes, compared to WT control (without LPS treatment).

### Analysis of cytokines, corticosterone, nitric oxide and plasma total cholesterol

The IL-8 secretion by HEK293 cells was analyzed in culture supernatants after a 20h incubation period in serum-free medium with or without BSA (2 mg/ml), utilizing an ELISA kit for human IL-8. Plasma levels of cytokines, corticosterone, cortisol, and nitrate (NOx) were quantified with corresponding ELISA or colorimetric kits. All samples and standards were measured in duplicate.

### Western blot analyses of MAPKs activity in WT and SR-BII expressing HEK293 cells

Wild type and hSR-BII-overexpressing HEK293 cells were grown in 6-well culture plates to confluence. Before the MAPKs activation assay, the cells were incubated for 6 hrs in serum-free DMEM. The cells were stimulated for varying periods of time with SAA (0.5ug/ml) at 37°C. After stimulation, the culture medium was immediately aspirated; the cells were placed on ice and washed three times with ice-cold PBS. Afterwards, the cells were scrapped into 100 μl of lysis buffer (25 mM Tris-HCl, pH 7.5, 1% NP-40, 0.5% (v/v) Triton X-100, 150uM NaCl and 1% (v/v) protease/phosphatase inhibitor cocktail (Thermo Fisher Scientific). After a 10-min incubation on ice the samples were centrifuged at 12,000 g for 10 min at 4°C. The cell extracts were collected and mixed with the 2× SDS sample buffer. The samples were separated on SDS-PAGE in 10% Tris-glycine pre-cast gels (Thermo Fisher Scientific) and then transferred to nitrocellulose membranes. After the transfer, the membranes were blocked with Tris-buffered saline containing 0.1% Tween 20 and 1% (w/v) nonfat dry milk and then probed with either one of three anti-phospho-MAPK antibodies or corresponding antibodies that recognize both active and inactive forms of each subfamily of MAP kinases, according to the manufacturer's protocols. The MAPK antibodies used in this study included anti-phospho-ERK1/2 (Thr^202^/Tyr^204^) antibody, anti-ERK1/2 antibody, anti-phospho-SAPK/JNK (Thr^183^/Tyr^185^) antibody, anti-SAPK/JNK antibody, anti-phospho-p38 MAPK (Thr^180^/Tyr^182^) antibody and anti-p38 MAPK antibody (Cell Signaling Technology). The immunoreactive bands were detected using an alkaline phosphatase-conjugated secondary antibody (Cell Signaling Technology) and chromogenic substrate for alkaline phosphatase (Invitrogen).

### Biomarkers of hepatotoxicity and histological analyses of hepatic and renal damage

Aspartate aminotransferase (AST) and alanine aminotransferase (ALT) activities in plasma were determined using corresponding colorimetric assay kits supplied by Sigma-Aldrich. For histological analyses formalin-fixed, paraffin-embedded 4 μm thin liver and kidney sections were stained with periodic acid-Schiff reagent (PAS) (Sigma Chemical Co.). Kidney histological changes were assessed in a blind manner in 10 different randomly selected 400X fields per animal from the cortex and 10 fields from the outer stripe of the outer medulla (OSOM). Kidney tubular damage was defined as tubular epithelial swelling, loss of brush border, vacuolar degeneration, necrotic tubules, cast formation, and desquamation. Liver damage was semi-quantitatively scored as previously described [45]: the amount of destruction of hepatic lobules, infiltration of inflammatory cells, hemorrhage, and hepatocyte necrosis was scored in 10 random fields per mouse and averaged. The score for each field was given according to the estimation of damage in each field. The degree of kidney and liver damage was estimated at 400X magnification by the following criteria: 1, 0 to 25%; 2, 25% to 50%; 3, 50% to 75%; 4, 75% to 100% of section showing any of the above mentioned signs of damage [45].

### Immunofluorescent microscopy

For immunofluorescent staining, specimens of liver tissue were embedded in OCT compound and frozen in a dry ice-acetone bath. The blocks were cut into 10 μM sections using a Leica CM 1900 cryostat and placed onto microscope slides. Sections were fixed with 3.7% formaldehyde for 10 min, washed 3 x 5 min with 0.5% Saponin in PBS and were blocked with 5% Goat Serum-0.05% Saponin-1% BSA-PBS for 1 h. Next, sections were incubated overnight at 4°C with rabbit antibodies against CD11b (cat. # NB110-89474, Novus Biologicals) followed by a 1h incubation with secondary antibodies conjugated with AlexaFluor 488 (Thermo Fisher Scientific). After two washes with PBS, sections were counterstained for nuclei with Hoechst 33342 (1μg/ml, Thermo Fisher Scientific), mounted using Vectashield antifade reagent (Vector, cat # H-1400), and visualized using Zeiss LSM 710 confocal microscope.

### Statistical analysis

Differences between the groups were examined for statistical significance by one-way analysis of variance (ANOVA). Alternatively, a two-tailed Student’s t-test was used. All data are expressed as mean values ± standard deviation (SD) with a p value of < 0.05 considered as significant.

## Results

### Uptake of Alexa Fluor 488-labeled SAA and SAA-induced secretion of IL-8 are increased in hSR-BI and hSR-BII- expressing vs. mock-transfected HeLa cells

**Fig 1:**
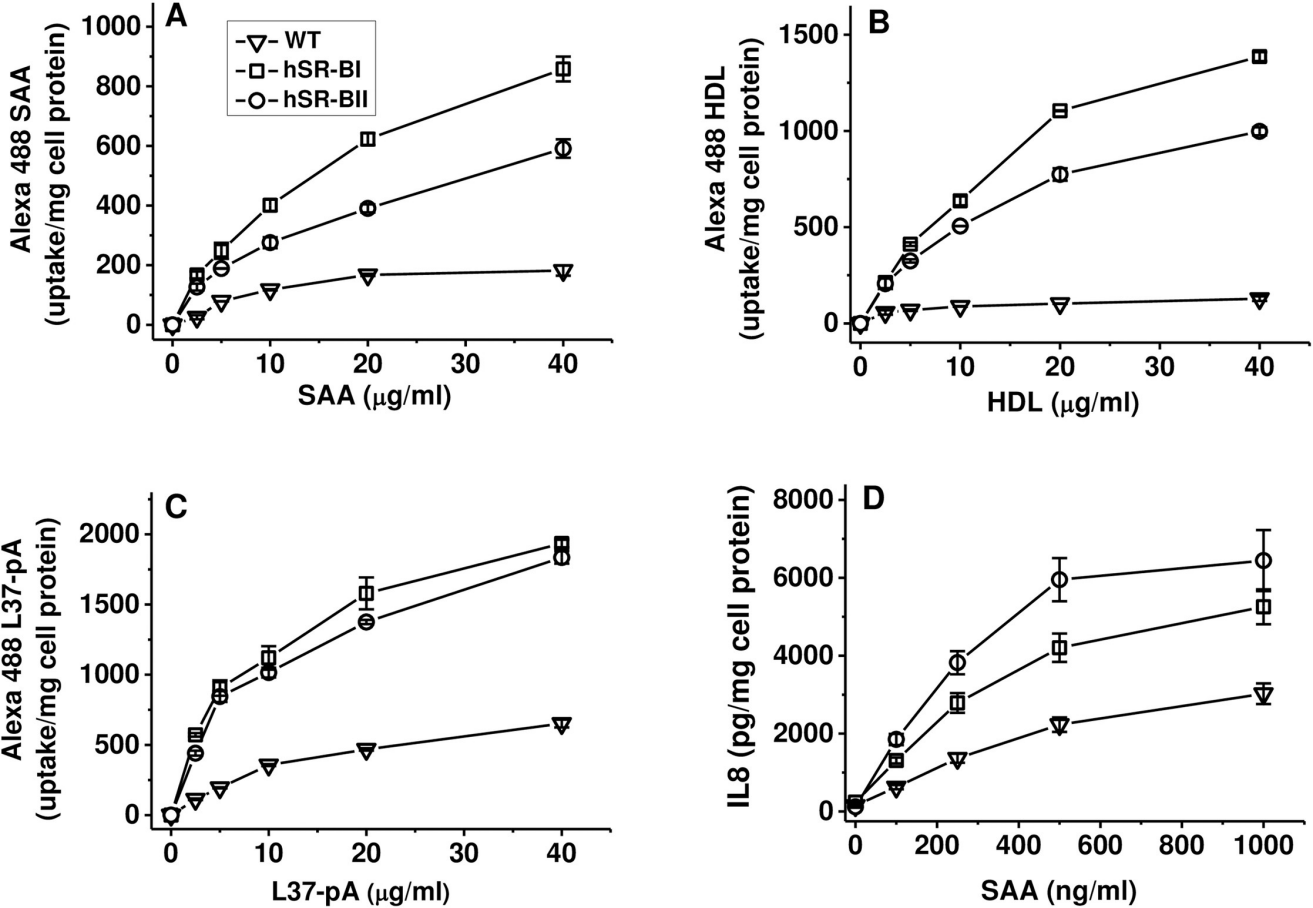
Dose-dependent SAA-induced IL-8 secretion and uptake of Alexa Fluor 488 ligands in mock-transfected, hSR-BI- and hSR-BII-expressing HeLa cells. (A) Mock-transfected and hSR-B-expressing cells were incubated with increasing concentrations of SAA for 20 h. IL-8 levels were determined in cell culture supernatants. (B-D) Cells were incubated with the indicated concentrations of Alexa Fluor 488 ligands for 2 h at 37°C without (total uptake) or in the presence of 100 μg/ml unlabeled ligands (nonspecific uptake). Cell-associated fluorescence was estimated using a fluorescence plate reader (see Materials and methods). Data represent one of three separate experiments that yielded similar results.

To test functional activity of hSR-BII as a potential SAA receptor, we measured cellular uptake of Fluor 488-labelled SAA using hSR-BII-expressing HeLa cells. Compared with mock-transfected cells, expression of hSR-BII markedly (~3-fold) increased the uptake of fluorescently labeled SAA (Fig 1A). Two other known ligands of SR-BII, HDL and L37pA, also demonstrated considerably higher uptake by hSR-BII–HeLa cells vs. control cells (Fig 1B, Fig 1C). Consistent with our earlier published data obtained by FACScan analysis [25] even higher increases in the uptake of all 3 fluorescently labeled ligands were found in hSR-BI-expressing cells vs. mock transfected cells.

SAA is a potent pro-inflammatory mediator capable to induce secretion of pro-inflammatory cytokines in cultured human phagocytic cells such as neutrophils [20,26] and THP-1 cells [23,48]. Our previous studies demonstrated that some of the proinflammatory activity of SAA could be mediated via hSR-BI [25] and CD36 [42]. To evaluate if hSR-BII-mediated SAA uptake could induce increases in cytokine production, we assessed levels of IL-8 secretion in hSR-BII-expressing HEK293 cells following stimulation with increasing doses of SAA (0, 0.1, 0.25, 0.5 and 1 μg/ml). After a 20 h treatment with SAA, we observed a 2-3-fold increase in IL-8 release in hSR-BII-HEK cells when compared with wild-type (WT) control cells (Fig 1D). A moderate (~2 fold) increase in IL-8 secretion level was also observed in hSR-BI-HEK cells when compared to control cells following SAA treatment.

### Competition of SR-B ligands with Alexa 488-SAA uptake in mock transfected, hSR-BI- and hSR-BII- expressing HeLa cells

**Fig 2:**
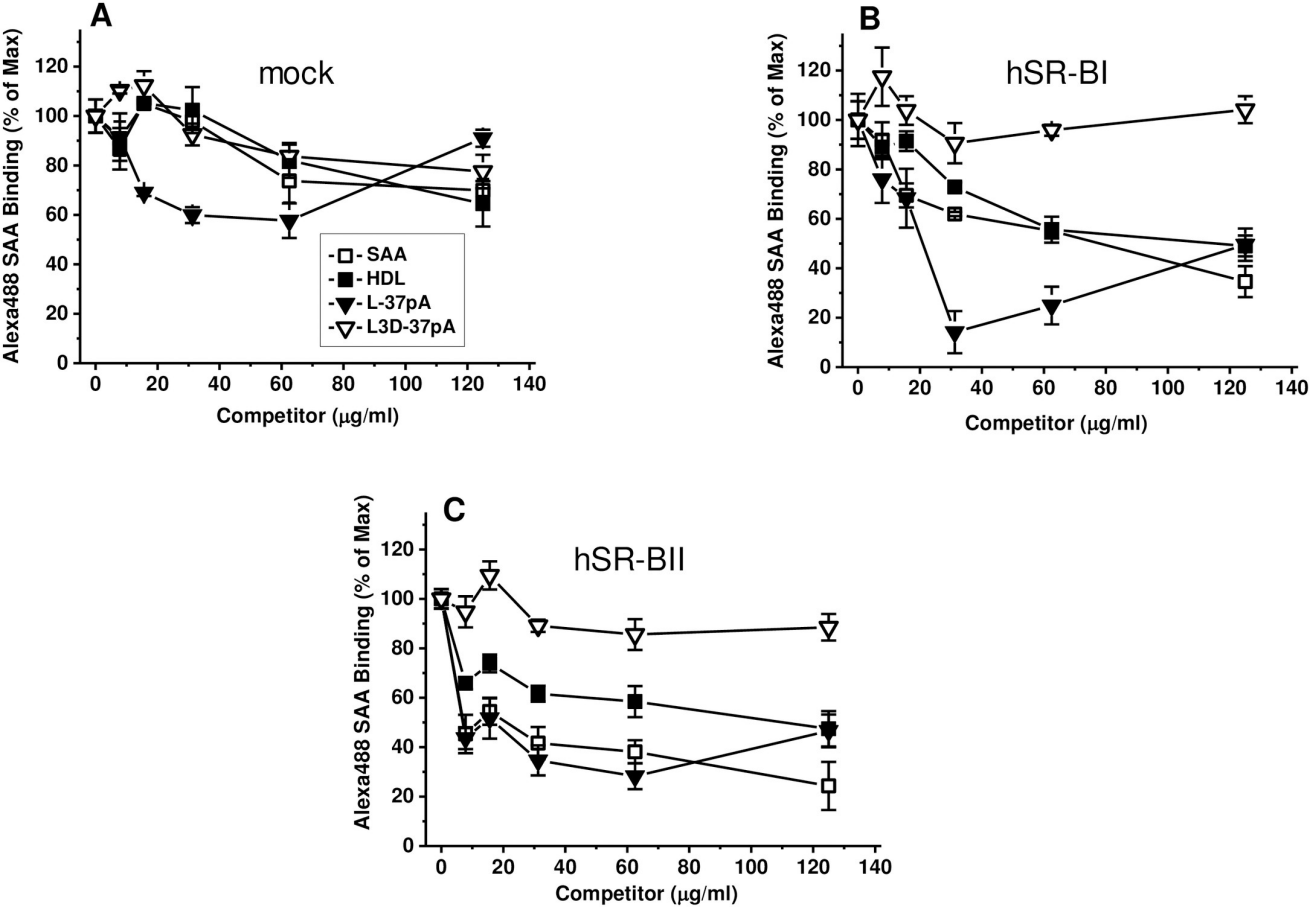
**Competition of SR-B ligands with Alexa Fluor 488 SAA uptake in mock-transfected (A), hSR-BI- (B) and hSR-BII-expressing (C) HeLa cells.** Cells were incubated with 5 μg/ml of Alexa Fluor 488 SAA with or without the indicated concentrations of unlabeled competitors for 2 h at 37°C. Unlabeled SAA was used as a control. Cell-associated fluorescence was estimated using a fluorescence plate reader and plotted as percentage of maximum binding (Max) in the absence (set as 100%) and in the presence of indicated competitor’s doses. Data represent one of three separate experiments that yielded similar results.

To test if hSR-BII is a potential SAA receptor we performed competition experiments using other well-known ligands of SR-Bs. As seen in Fig 2B and 2C, HDL and L37pA efficiently competed with Alexa 488 SAA in a dose-dependent manner in both hSR-BI- and hSR-BII-expressing cells. Unlabeled SAA also potently inhibited uptake of Alexa 488 SAA by as much as 65% and 80% in hSR-BI and hSR-BII-expressing cells, respectively. No competition was found with the L3D-37pA control peptide (Fig 2A), which contains three D-amino acid substitutions and was previously shown to be poor ligand for SR-Bs [35].

### Effects of SAA treatment on MAPKs activation in WT and hSR-BII-expressing HEK 293 cells

**Fig 3:**
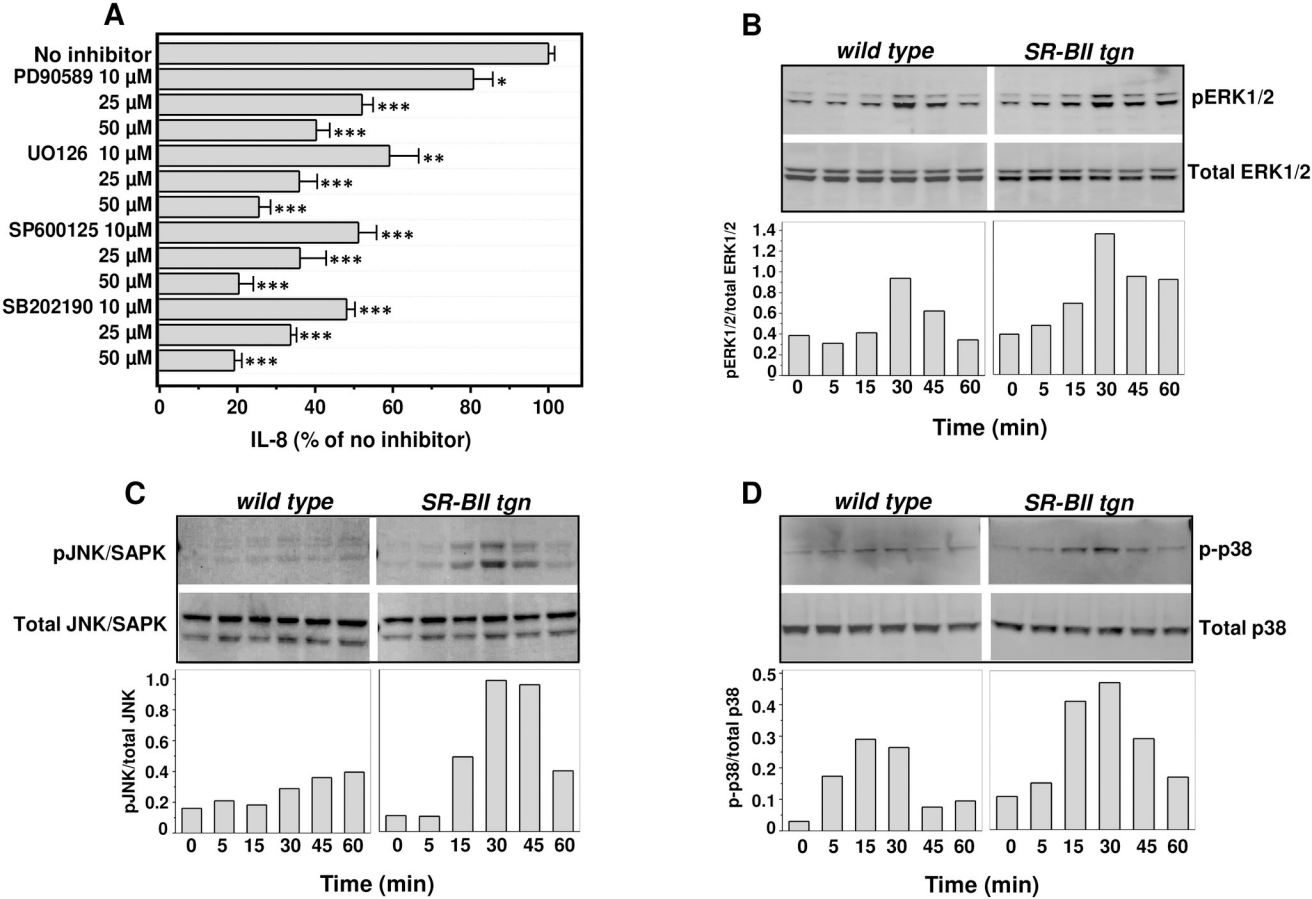
Evaluation of MAPKs contribution to hSR-BII-dependent inflammatory signaling induced by SAA. (A) Effects of specific MAPK inhibitors on SAA-induced IL-8 secretion in hSR-BII-expressing HEK293 cells. Cells were treated with the indicated doses of MAPKs inhibitors for 1 h prior to stimulation with SAA (0.5 μg/ml). Levels of the secreted IL-8 were measured in cell culture supernatants after 20 h. Data are presented as means ± S.D. of one of three separate experiments performed in duplicates that yielded similar results. (B-D) Western blot analysis of SAA-induced MAPKs phosphorylation in wild type and hSR-BII-expressing HEK293 cells. Cells were treated with 0.5 μg/ml of SAA for the indicated time intervals. The expression of non-phosphorylated forms of MAPKs is shown as the loading control. The resulting bands were quantified using GeneTools image analysis software (Syngene). The data are presented as the ratio of integral optic density for phosphorylated MAPK bands to the corresponding integral optic density values for total MAPK bands. The data represent one of two separate experiments that yielded similar results. * p<0.05, ** p<0.01, *** p<0.005 *vs*. IL-8 levels in the absence of any inhibitor.

Our previous study demonstrated that the hSR-BI-dependent pro-inflammatory response in HeLa cells induced by SAA involves activation of 2 mitogen-activated protein kinases—ERK1/2 and p38 [25]. To investigate whether the MAPK family kinases contribute to the increased SAA-induced IL-8 release found in hSR-BII cells, selective inhibitors of each MAPK signaling pathway were tested in WT and hSR-BII-expressing HEK293 cells. Our experiments revealed that all three MAPKs inhibitors potently, though to a different extent, blocked the SAA-induced IL-8 release in hSR-BII-expressing cells in a dose-dependent manner (Fig 3A). In order to further investigate the direct contribution of each MAPK in SAA-induced hSR-BII-dependent signaling, we assessed the levels of ERK1/2, JNK, and p38 phosphorylation in hSR-BII-expressing and control HEK293 cells following SAA stimulation. As shown in Fig 3, upon cell treatment with SAA (0.5 μg/ml) for 0–60 min, all three MAPKs were transiently phosphorylated in both cell types; however, hSR-BII-expressing cells demonstrated markedly higher levels of JNK (~2.5–3 fold) phosphorylation with moderately increased phosphorylation of ERK1/2 (by ~ 45–60%) and p38 (by ~ 45–70%), when compared with control WT cells (Fig 3B–3D).

### Effects of acute SAA administration on plasma pro-inflammatory cytokine and NO levels in wild type, hSR-BI and hSR-BII transgenic mice

**Fig 4:**
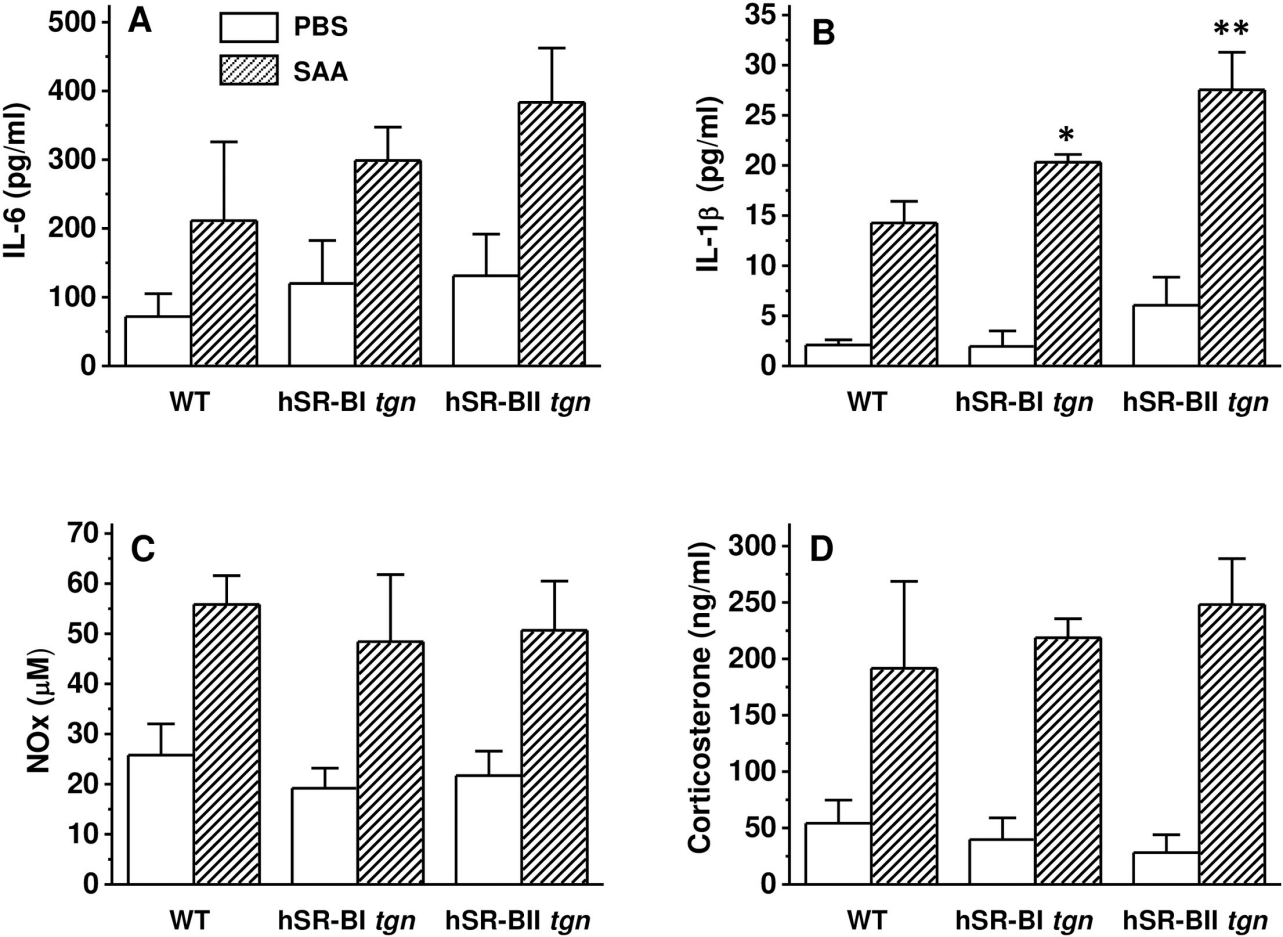
Plasma levels of cytokines, nitrite/nitrate (NOx) and corticosterone in WT, hSR-BI tgn and hSR-BII tgn mice injected with SAA. SAA (2 mg/kg, IP) or PBS was injected into WT, hSR-BI and hSR-BII tgn mice. Six hours after the SAA injection, mice were euthanized for plasma and organ collection. Plasma levels of IL-6 (A), IL-1β (B) and corticosterone (D) were determined by ELISA, and plasma NOx levels were measured using a colorimetric kit. Values are the mean ± SD (n = 5). * p<0.05, ** p<0.01, vs. WT SAA-treated levels.

To investigate the influence of each splice variant’s (hSR-BI and hSR-BII), on SAA-induced pro-inflammatory activity *in vivo*, control WT, transgenic hSR-BI and hSR-BII mice were injected with PBS or SAA and inflammatory responses were assessed by measuring pro-inflammatory cytokines and nitric oxide (NO) serum levels 6 hours following injection. In order to avoid non-physiological effects of lipid-poor recombinant SAA reported by Christenson et al [49], our study mice received intraperitoneal administration of recombinant SAA. Six hours after the injection, all SAA protein in plasma samples analyzed in non-reducing conditions by Western Blot assay using an anti-SAA antibody was found associated with HDL (data not shown). All SAA-treated animals (both WT and transgenic) demonstrated a modest (~ 3–5 –fold) increase in plasma levels of both IL-6 (Fig 4A) and IL-1β (Fig 4B), although only IL-1β levels were moderately (by 1.5 and 1.8-fold, respectively) elevated in hSR-BI and hSR-BII mice, vs. WT mice. All mice had similar ~ 2-fold increase of NO plasma levels following SAA administration (Fig 4C). Following SAA injection we observed robust increases of plasma corticosterone levels in all groups of mice (Fig 4D), indicating that hSR-BI and hSR-BII transgenic mice, despite their markedly reduced HDL-cholesterol plasma levels [45], can still respond adequately to SAA-triggered inflammation by the release of glucocorticoids.

### Liver and kidney mRNA expression of pro-inflammatory markers in response to SAA is increased in hSR-BI and hSR-BII transgenic mice

**Fig 5:**
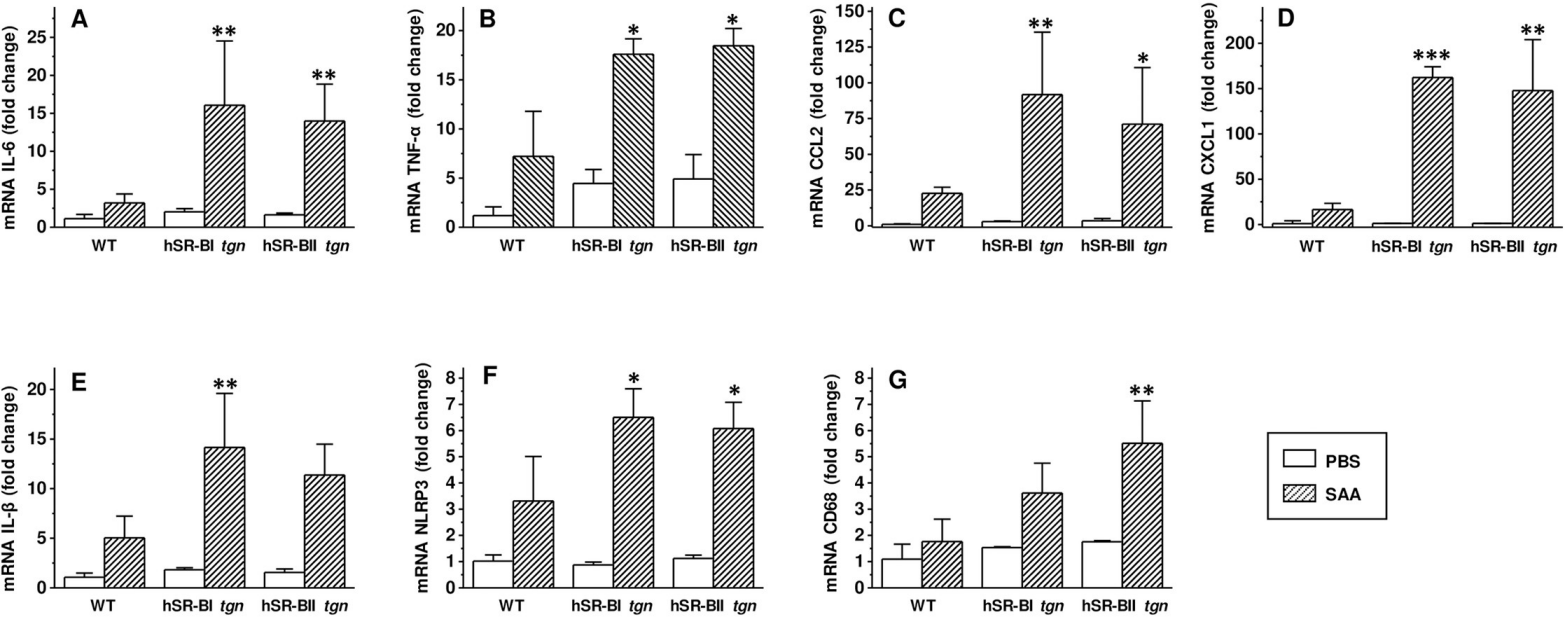
Hepatic gene expression of inflammatory markers in WT, hSR-BI and hSR-BII transgenic mice injected with SAA. SAA (2 mg/kg, IP) or PBS was injected into WT, hSR-BI tgn and hSR-BII tgn mice. Six hours after the SAA injection, mice were euthanized and liver tissue was collected for mRNA extraction and qRT-PCR as described in Materials and methods. Expression levels of IL-6 (A), TNF-α (B), CCL2 (C), CXCL1 (D), IL-1β (E), NLRP3 (F), and CD68 (G) were normalized by GAPDH and presented as the fold change relative to PBS-treated control. Values shown are the mean ± SD (n = 3, for PBS-treated groups, n = 5 for SAA-treated groups). * p<0.05, ** p<0.01, *** p<0.005 *vs*. WT SAA-treated mice.

**Fig 6:**
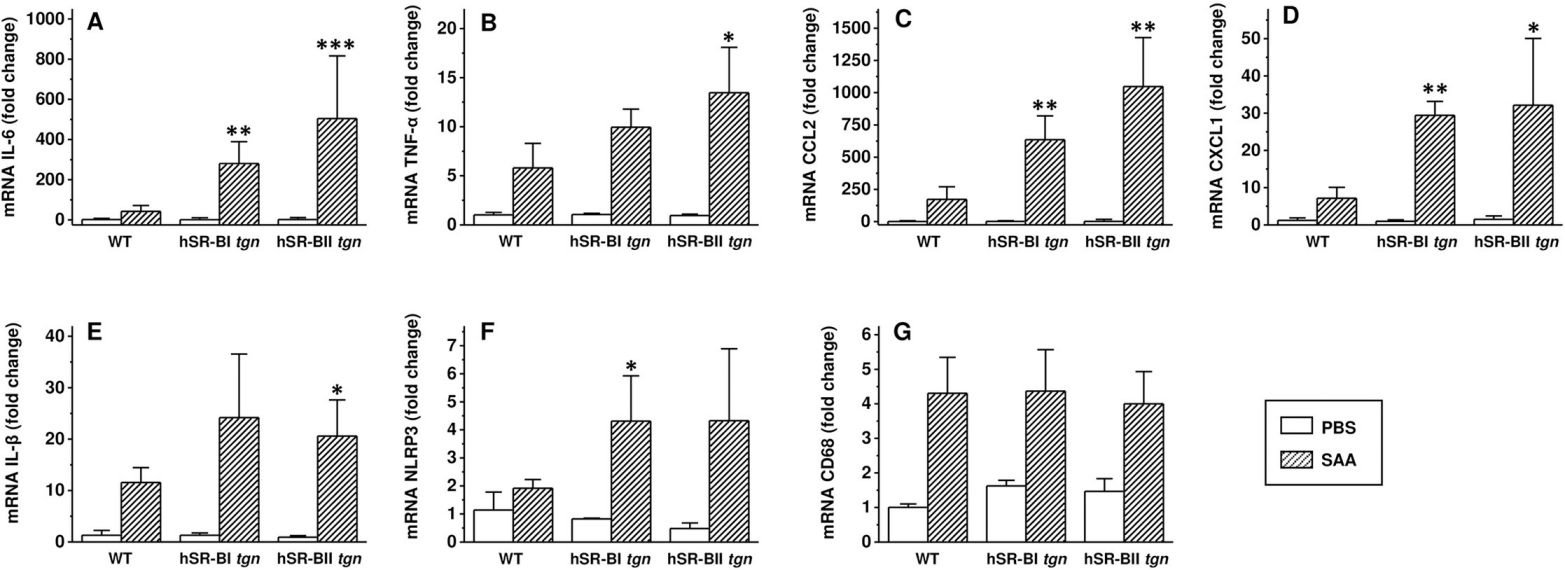
Kidney gene expression of inflammatory markers in WT, hSR-BI and hSR-BII transgenic mice injected with SAA. SAA (2 mg/kg, IP) or PBS was injected into WT, hSR-BI and hSR-BII tgn mice. Mice were euthanized after 6 hours; kidney samples were collected and used for mRNA extraction and qRT-PCR as described in Materials and methods. Expression levels of IL-6 (A), TNF-α (B), CCL2 (C), CXCL1 (D), IL-1β (E), NLRP3 (F), and CD68 (G) were normalized by GAPDH and presented as the fold change relative to PBS-treated control. Values shown are the mean ± SD (n = 3, for PBS-treated groups, n = 5 for SAA-treated groups). * p<0.05, ** p<0.01, *** p<0.005 *vs*. WT SAA-treated mice.

To investigate if any inflammation associated changes have occurred locally, hepatic and renal tissues of SAA- and PBS-treated mice were assessed for the gene expression of several pro-inflammatory mediators by quantitative PCR six hours after SAA or PBS injection. Despite the relatively low increase of systemic plasma cytokines to acute SAA injection, we detected a very strong increase of all tested pro-inflammatory markers in both organs. Hepatic expression of all cytokines (except for IL-1β, that was significantly higher only in hSR-BI *vs*. WT mice, Fig 5E) was markedly higher in hSR-BI and hSR-BII transgenic mice than in WT mice treated with SAA, with the most dramatic (~ 4–8 fold) increases observed in IL-6, CCL2 and CXCL1 levels (Fig 5A, 5C and 5D). We have also found moderately increased (~ 2 times) expression of inflammasome-related NLRP3 gene (Fig 5F) in both hSR-BI and hSR-BII mice, and macrophage marker CD68 was markedly higher (~ 2.5 times) in the livers of hSR-BII transgenic mice compared to WT mice (Fig 5G). In the kidneys (Fig 6), a similar pattern of increased pro-inflammatory markers gene expression was found: CXCL1 and CXCL2 gene expression was significantly higher in kidneys from both hSR-BI and hSR-BII transgenic mice treated with SAA, while TNFα (Fig 6B) and IL-1β (Fig 6E) mRNA levels were significantly increased only in kidneys of hSR-BII mice, and NLRP3 gene expression (Fig 6F) was significantly increased only in kidneys of hSR-BI mice, when compared to WT mice. Renal CD68 expression (Fig 6G) increases were similar in all mice (WT and hSR-B transgenic) treated with SAA. There was no significant difference in SAA-induced cytokine expression in the liver or kidney between hSR-BI- and hSR-BII transgenic mice, although, in the kidneys, there was a tendency towards higher expression of some cytokines in hSR-BII *versus* hSR-BI mice.

### Plasma levels of liver injury markers are higher in hSRB transgenic mice vs. wild type mice following acute SAA injection

**Fig 7:**
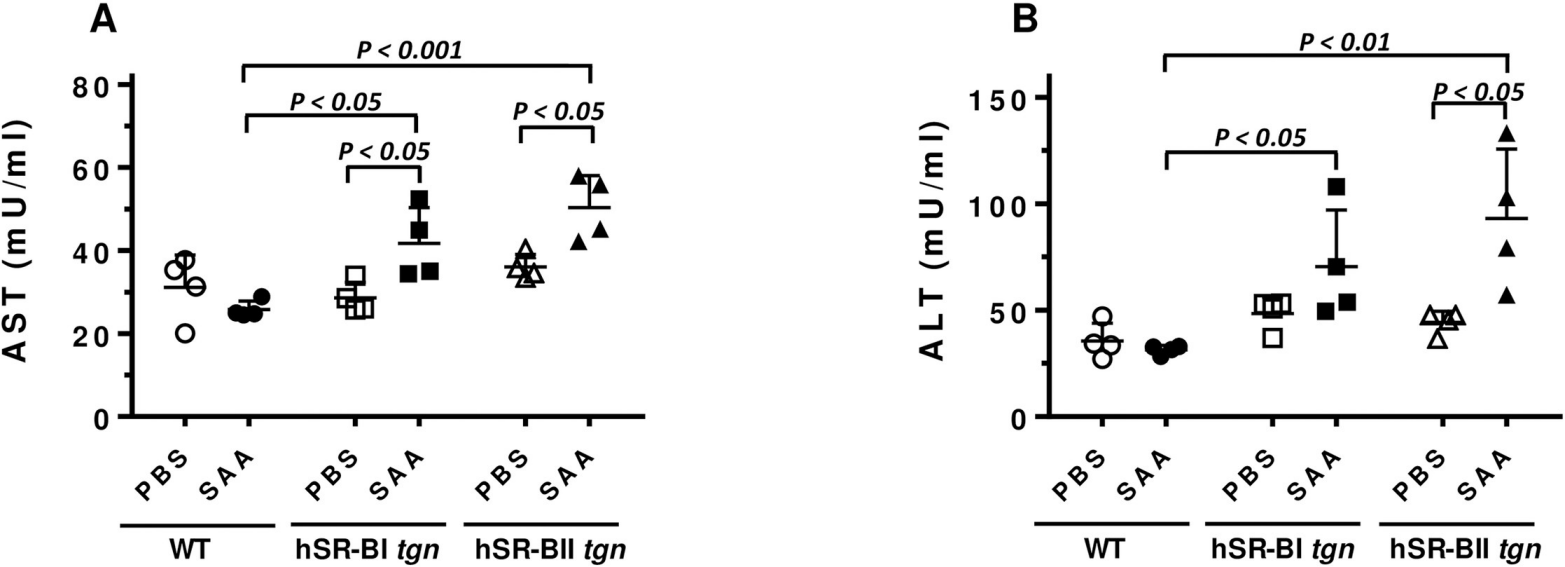
Effect of acute SAA injection on plasma activity of transaminases in WT, hSR-BI and hSR-BII transgenic mice. Six hours after PBS or SAA IP injection mice were euthanized and blood was collected for AST (A) and ALT (B) activity assays (n = 4 for each PBS- and SAA-treated groups).

To assess SAA-induced liver damage, activity of plasma liver-specific enzymes, alanine aminotransferase (ALT) and aspartate aminotransferase (AST), were measured 6 hours after SAA injection. We observed a moderate, but statistically significant, increase of AST activity in plasma of hSR-BI (~by 45%) and hSR-BII (~by 40%) transgenic mice, while no changes in AST activity were found in plasma of wild type mice (Fig 7A). Of all groups, only hSR-BII transgenic mice demonstrated statistically significant, (~ 2-fold) increase in plasma ALT activity following SAA injection (Fig 7B).

### Histological and immunofluorescent analyses of SAA-induced liver and kidney injury of WT, hSR-BI and hSR-BII transgenic mice

**Fig 8:**
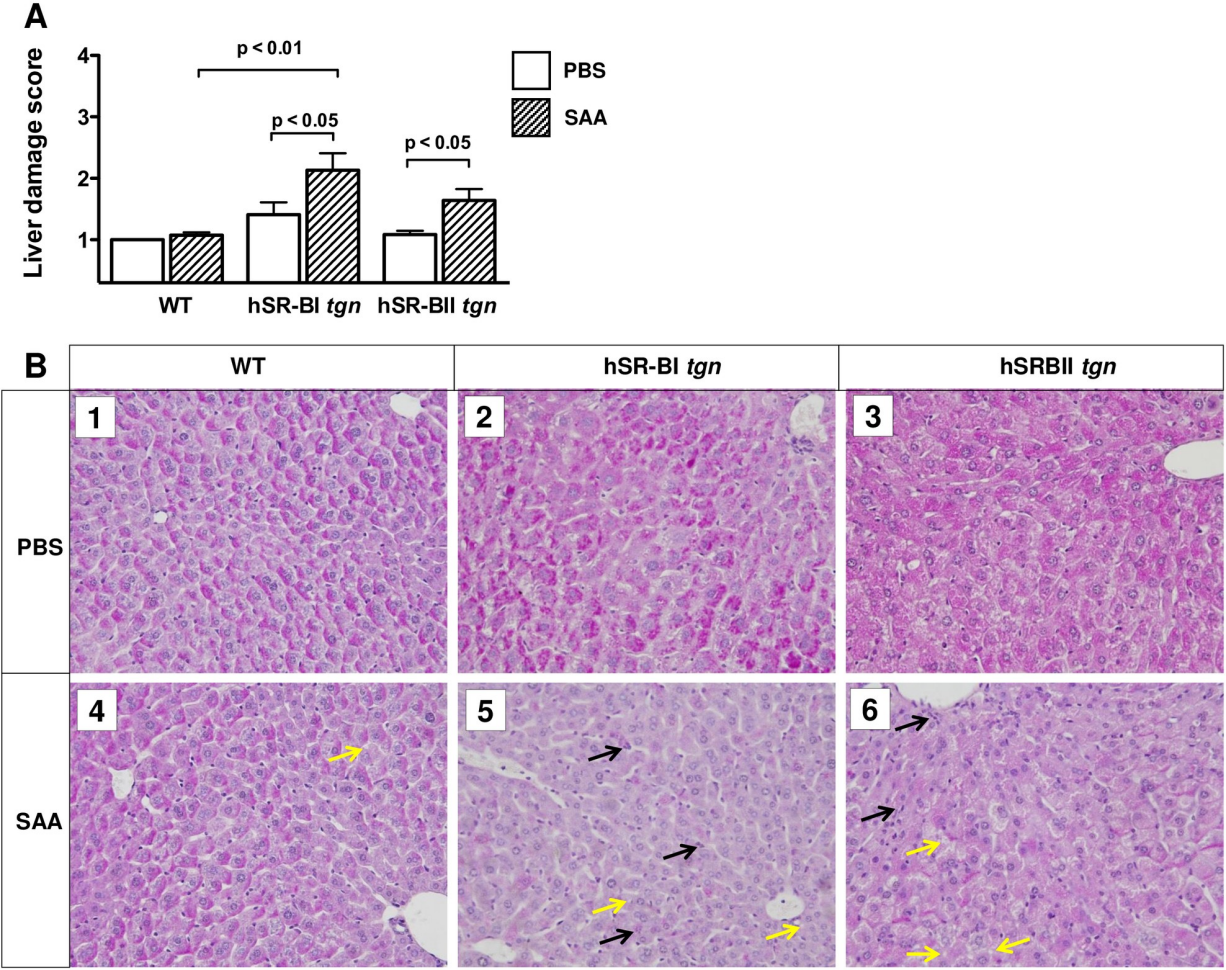
SAA-induced histological liver damage in various mice. (A) Semi-quantitative histological analysis of liver injury. Liver injury was defined as the amount of destruction of hepatic lobules, presence of microvacuolization, infiltration of inflammatory cells, hemorrhage, and hepatocyte necrosis, and scored from 1 through 4 according to % area of involvement per HPF (400X). Liver damage scores for mice that received PBS (n = 4-7/group, open bars) or an SAA injection (n = 5/group, dashed bars). (B) Representative images (400X) of liver sections stained by PAS from each group (mice that received PBS: WT—B1, hSR-BI tgn–B2, and hSR-BII tgn–B3), mice that received SAA: WT B4, hSR-BI tgn—B5, and hSR-BII tgn—B6). Microvacuolization of hepatocytes is labeled with yellow arrows, and inflammatory cell infiltration is labeled with black arrows. Values shown as mean ± SD (n = 3, for PBS-treated groups, n = 5 for SAA-treated groups); data analyzed by one-way ANOVA with Bonferoni’s post-test; p values are described in the graph.

Histological changes were examined in the livers and kidneys of mice 6 hours following PBS or SAA injection via the optical microscopy. As shown in Fig 8A and 8B (panels 1–3), WT, hSR-BI tgn, and hSR-BII tgn mice that received PBS had liver histology scores that were not statistically different. Six hours after SAA injection hSR-BI (Fig 8A and 8B, panels 5 and 6) mice developed statistically significant histological liver damage in comparison to SAA-treated WT mice, which did not show any liver or kidney histological damage after SAA injection. There were no statistical differences between SAA-treated hSR-BI and hSR-BII transgenic mice regarding histological scores. The most notable on optical microscopy histological damage was due to microvacuolization of hepatocytes (yellow arrows) and inflammatory cell infiltration (black arrows). In the kidneys, SAA injection, at the dose studied, did not induce any significant histological damage beyond very rare tubular vacuolization in all mice. Immunofluorescent staining of liver sections from PBS- and SAA-treated mice using a CD11b antibody, known to recognize various cells of myeloid lineage, including monocytes, macrophages and neutrophils, revealed a markedly higher presence of CD11b+ cells in both hSR-BI and hSR-BII tgn mice compared to WT mice subjected to SAA injection (S1 Fig).

## Discussion

Serum amyloid A is an endogenous damage-associated molecular pattern (DAMP) molecule which, when recognized by the host, initiates tissue-controlled immune responses [50]. SAA levels in blood were shown to be markedly elevated during various chronic inflammatory conditions [8–11]; however, SAA-specific pathogenic roles and the potential mechanisms of its contribution to these pathological states are not completely understood.

To date several receptors, including FPRL-1 [21], TLR2 [26,51] and TLR4 [28], receptor for advanced glycosylation end products (RAGE) [44], as well as class B scavenger receptors, SR-BI and CD36 [24,25,42], have been identified as SAA receptors that mediate its various functions. Our previous findings revealed that SR-BI and CD36, the most well–characterized members of SR-B family, could function as SAA receptors mediating its uptake and signaling in epithelial cells overexpressing these two proteins. Other recent studies provided additional evidence supporting SR-BI role as a mediator of diverse SAA effects. Mullan et al. [43] have shown that pro-inflammatory responses of SAA closely correlate with SR-BI expression on RA FLCs (fibroblast-like cells) *in vitro*, and SAA-induced cytokine production in human microvascular endothelial cells can be down-regulated by SR-BI antagonists, ApoA-I mimetic peptides, and a specific anti–SR-B1 antibody. Hong et al [52] demonstrated that blockage of SR-BI and p38MAPK inhibited SAA-induced cell proliferation, migration and tube formation in human vein endothelial cells (HUVEC).

SR-BII, a splice variant of SR-BI, another member of SR-B family, is known mainly as a lipoprotein receptor involved with cholesterol transport, whereas its role as a receptor for pathogens, including various bacterial products, and danger-associated ligands, such as SAA, has not received proper attention until recently. In our opinion, SR-BII physiological importance was underestimated by the researches in particular because most studies failed to detect its expression when commercial LIMP2 sequence-based antibody was mistakenly used instead of anti-SR-BII antibody [53]. Our recent studies using custom anti-SR-BII antibodies, demonstrated SR-BII expression in the human liver and isolated human hepatocytes by both Western blot analysis and immunofluorescent assay (data not shown).

Our earlier studies demonstrated that all three SR-B family members, SR-BI, SR-BII and CD36, are able to recognize and mediate pro-inflammatory signaling of bacterial products, such as LPS and GroEL, suggesting their important roles in innate immunity and host defense [35]. In this study we tested the potential role of SR-BII as a SAA receptor involved in its uptake and signaling, utilizing hSR-BII expressing epithelial cells lines. Additionally, using hSR-BI- and hSR-BII-overexpressing transgenic mice subjected to acute SAA injection we investigated whether hSR-BI and hSR-BII could also contribute to SAA-induced pro-inflammatory effects *in vivo*.

The results of this study provide new evidence that hSR-BII may function as a SAA receptor, involved in its uptake and pro-inflammatory signaling. Our data demonstrate that both hSR-BI- and hSR-BII-expressing HeLa cells have significantly increased (4- and 6- fold, respectively) Alexa Fluor 488-SAA uptake when compared to mock-transfected control cells. The specificity of SAA uptake by hSR-BII was further confirmed in competition experiments, where non-labeled SAA as well as two other SR-B ligands, HDL and L37pA, were shown to efficiently block Alexa Fluor 488 SAA uptake in both hSR-BI and hSR-BII- expressing HeLa cells, whereas L37pA-3D peptide had no inhibitory effect. In addition to the enhanced uptake of SAA via the hSR-BII in HeLa cells we also found significant (~3–3.5-fold) increase in SAA-induced pro-inflammatory cytokine IL-8 secretion in hSR-BII-expressing HEK293 cells.

MAPK signaling pathways have been implicated in SAA-induced pro-inflammatory cytokine/chemokine production in several cell types including neutrophils [22], monocytes [54] endothelial [52] and epithelial cells [25,42]. Previously we demonstrated that phosphorylation of ERK1/2 and p38 MAPKs was significantly increased in hSR-BI-expressing cells following SAA treatment, implying hSR-BI contribution to SAA-induced MAPK-mediated pro-inflammatory signaling [25]. No data have been previously reported regarding the SR-BII functioning as a signal transducing receptor. However, similarly to SR-BI, in some cell types SR-BII has been localized to plasma membrane caveolae [47], specialized microdomains widely implicated in signaling processes. Additionally, six proline-rich motifs (known to bind SH3-domain-containing signaling proteins) and a SH2-binding site, identified in the C-terminal cytoplasmic tail of hSR-BII [55], could be responsible for the signaling potential of this receptor. Using pharmacological blockers of each MAPK signaling pathways, we found that SAA-induced IL-8 release could be efficiently blocked by each tested specific signaling inhibitor in SR-BII-expressing cells. The results of the MAPK activation assays demonstrated that SAA treatment resulted in higher phosphorylation of all MAPKs in hSR-BII-expressing cells when compared to WT control cells, with the most noticeable (2.5–3 fold) difference observed for JNK MAPK. Thus, our data suggests that hSR-BII-dependent SAA-induced pro-inflammatory signaling could potentially activate each of three MAPK signaling cascades. Further studies are needed to identify which upstream signaling mediators lead to MAPKs activation following SR-BII-SAA interaction. As potential candidates for this role we suggest Src family non-receptor tyrosine kinases, known as important upstream regulators of MAPKs [56–59] and possessing both SH3 and SH2 domains in their molecules [60].

Utilizing hSR-BI- and hSR-BII-transgenic mice as gain-of-function models allowed us to access the potential in vivo contribution of both receptors to SAA-induced acute inflammation. The dose of SAA used in this study (2mg/kg) corresponded to SAA levels found during some mild chronic inflammatory conditions, with estimated plasma SAA to HDL ratio 1:20 (w/w). To assess the potential role of hSR-BI and hSR-BII as mediators of SAA-induced inflammatory response *in vivo*, we compared several pro-inflammatory markers levels both in plasma and gene tissue expression in liver and kidney of SR-B transgenic and wild-type mice 6 hours following SAA administration. SAA treatment caused modest responses for all measured plasma pro-inflammatory markers that were not statistically different between the groups of animals. Although the SAA dose used in this study exceeded the normal range of plasma SAA levels, it was considerably lower than those observed at pathological inflammatory conditions. SAA-treated mice were exposed to only one injection of SAA while in patients with chronic inflammatory conditions are continuously exposed to SAA for a much longer period of time. Additionally, the pro-inflammatory potential of SAA could be reduced due to its association with plasma lipoproteins. Therefore, we did not expect to see any significant changes in systemic inflammatory markers in our experimental setting. Despite the relatively low systemic pro-inflammatory response, all groups of SAA-treated mice demonstrated significant release of plasma corticosterone, reaching levels that are typically found during more severe inflammation.

We also assessed the effects of acute SAA injection on tissue expression of several genes, related to inflammation and cell adhesion, in hSR-B transgenic and wild type mice. We found that gene expression of all inflammation-associated mediators measured in liver and kidney was markedly up-regulated in all SAA-treated groups of mice. However, both hepatic and renal expression of almost all pro-inflammatory markers was significantly higher in SAA-treated hSR-BI and hSR-BII transgenic mice than in WT mice. Importantly, the strongest increases were found for both pro-inflammatory chemokines, CXCL1 and CCL2. Earlier *in vitro* and *in vivo* studies demonstrated important role played by SAA in chemotaxis of human monocytes and polymorphonuclear leukocytes [18]. Apparently, this intrinsic chemoattractant activity of SAA could be significantly enhanced by the rapid induction of cooperating chemokines in migrating macrophages, further escalating the pro-inflammatory cascade.

It has been suggested that at local sites of inflammation, upon release of proteolytic enzymes by activated monocytes [61] or leukocytes [62], free SAA is released from its complexes with HDL, creating a concentration gradient and inducing recruitment of inflammatory cells, leading to further augmentation of local inflammatory processes [18]. We found that, in addition to the increased cytokine/chemokine expression, SAA-treated hSR-BII mice had ~ 2.5 fold higher (compared to wild type) hepatic expression of CD68, which is known to be highly expressed on activated monocytes and macrophages, including Kupffer cells [63–65] and is commonly used as a marker of macrophage activation and migration [66,67]. Hepatic and renal expression of another inflammatory marker, the inflammasome-related NLRP3 gene was also found to be moderately (2-fold) higher in SAA-treated hSR-B transgenic mice as compared to WT mice. Recent findings implicated SAA as a potent activator of the NLRP3 inflammasome-associated signaling cascade resulting in activation of caspase-1 and IL-1β secretion [68] via the ATP receptor P2X7, although the specific mechanisms involved in this pathway activation have not been completely investigated. Our data suggests that hSR-B receptors may contribute to the SAA-induced inflammasome-mediated inflammatory response by facilitating SAA uptake into the target cells. Consistent with the higher SAA-induced pro-inflammatory response observed in the liver of hSR-B transgenic mice, histology scores reflecting the extent of liver damage after SAA challenge were found to be increased (~ 1.5-fold) only in hSR-BI and hSR-BII transgenic mice. Increased activity of plasma transaminases, AST and ALT, highly sensitive and specific markers of hepatotoxicity, found in hSR-BI and hSR-BII transgenic mice, but not in the wild type mice, following SAA injection, further supports a substantial role of hSR-B receptors in SAA-induced liver injury. Furthermore, the increased number of CD11b positive cells in the livers of SAA-treated hSR-BI and hSR-BII transgenic mice vs. wild type mice, demonstrated using immunofluorescent microscopy, provides additional evidence of hSR-B-dependent inflammatory cell infiltration induced by acute SAA injection. No appreciable kidney damage was found in either group of mice following SAA treatment; this finding may be due to insufficient SAA exposure reflecting different timing/sensitivity of SAA-induced damage in different organs.

Recently our group described the roles of SR-BI and SR-BII as important receptors for lipopolysaccharide (LPS): hSR-BI and hSR-BII transgenic mice subjected to IP LPS injection exhibited increased systemic inflammation, increased hepatic and renal expression of inflammation–related genes, and more importantly, more liver and kidney histological lesions than LPS-treated WT mice [45]. While the changes described in the current study, especially regarding histological damage, were milder than those associated with the LPS challenge, now we were able to demonstrate that SAA, an inflammatory protein commonly found in several chronic conditions, signals through SR-BI and SR-BII receptors.

In conclusion, we found that human SR-BII, a splice variant of hSR-BI, is a functional receptor of SAA, capable of mediating its uptake and pro-inflammatory signaling. The *in vivo* studies revealing higher SAA-induced pro-inflammatory responses along with moderate liver damage in hSR-BI- and hSR-BII-transgenic mice further highlight the important role of the class B scavenger receptor family as mediators of PAMP- and DAMP-induced inflammation and support SR-BI/BII’s potential contribution to the host immune response.

## Supporting information

S1 Fig Identification of CD11b+ cells by immunofluorescent microscopy in liver frozen sections from various groups of mice. Frozen liver sections from PBS-treated (panels 1–3) and SAA-treated (panels 4–6) mice were stained using an anti-CD11b antibody, followed by the Alexa 488 Fluor-conjugated secondary antibody (green), according to the protocol described in Material and Methods. Hoechst 33342 nucleic counterstain appears blue. Scale bars, 50 μM.
(TIF)

## Abbreviations

ALT: alanine aminotransferase
AST: aspartate aminotransferase
CXCL1: chemokine (C-X-C motif) ligand 1
CS: corticosterone
ERK1/2: extracellular signal-regulated protein kinase 1 and 2
GAPDH: glyceraldehyde-3-phosphate dehydrogenase
GCs: glucocorticoids
HPF: high-power field
IM: intramuscular
JNK: c-Jun N-terminal kinase
MAPK: mitogen-activated protein kinases
NLRP3: NLR family, pyrin domain containing 3
SAA: serum amyloid A
SR-BI-KO: scavenger receptor BI knockout mice
hSR-BI tgn: human scavenger receptor BI transgenic mice
hSR-BII tgn: human scavenger receptor BII transgenic mice
SFKs: Src-family kinases
WT: wild type mice
