# Supplementary figures and images for: Human SR-BII mediates SAA uptake and contributes to SAA pro-inflammatory signaling in vitro and in vivo
Source: PLoS One. 2017 Apr 19;12(4):e0175824. doi: 10.1371/journal.pone.0175824 (PMC5396919; doi:10.1371/journal.pone.0175824)

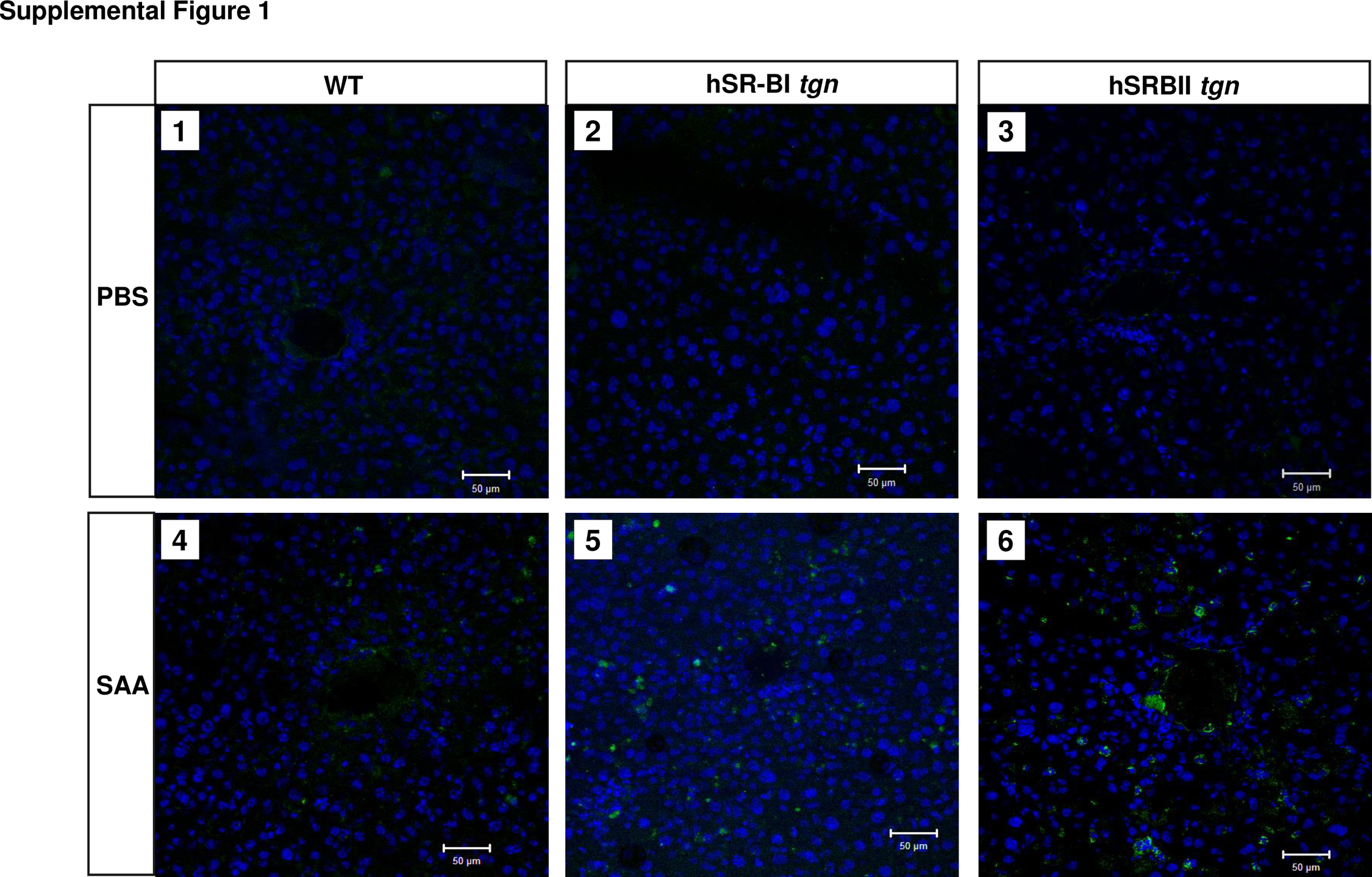

Supplement: S1 Fig — Frozen liver sections from PBS-treated (panels 1–3) and SAA-treated (panels 4–6) mice were stained using an anti-CD11b antibody, followed by the Alexa 488 Fluor-conjugated secondary antibody (green), according to the protocol described in Material and Methods. Hoechst 33342 nucleic counterstain appears blue. Scale bars, 50 μM. (TIF) [file pone.0175824.s001.tif]
